# Supplementary figures and images for: The SARM1 TIR domain produces glycocyclic ADPR molecules as minor products
Source: PLoS One. 2024 Apr 18;19(4):e0302251. doi: 10.1371/journal.pone.0302251 (PMC11025887; doi:10.1371/journal.pone.0302251)

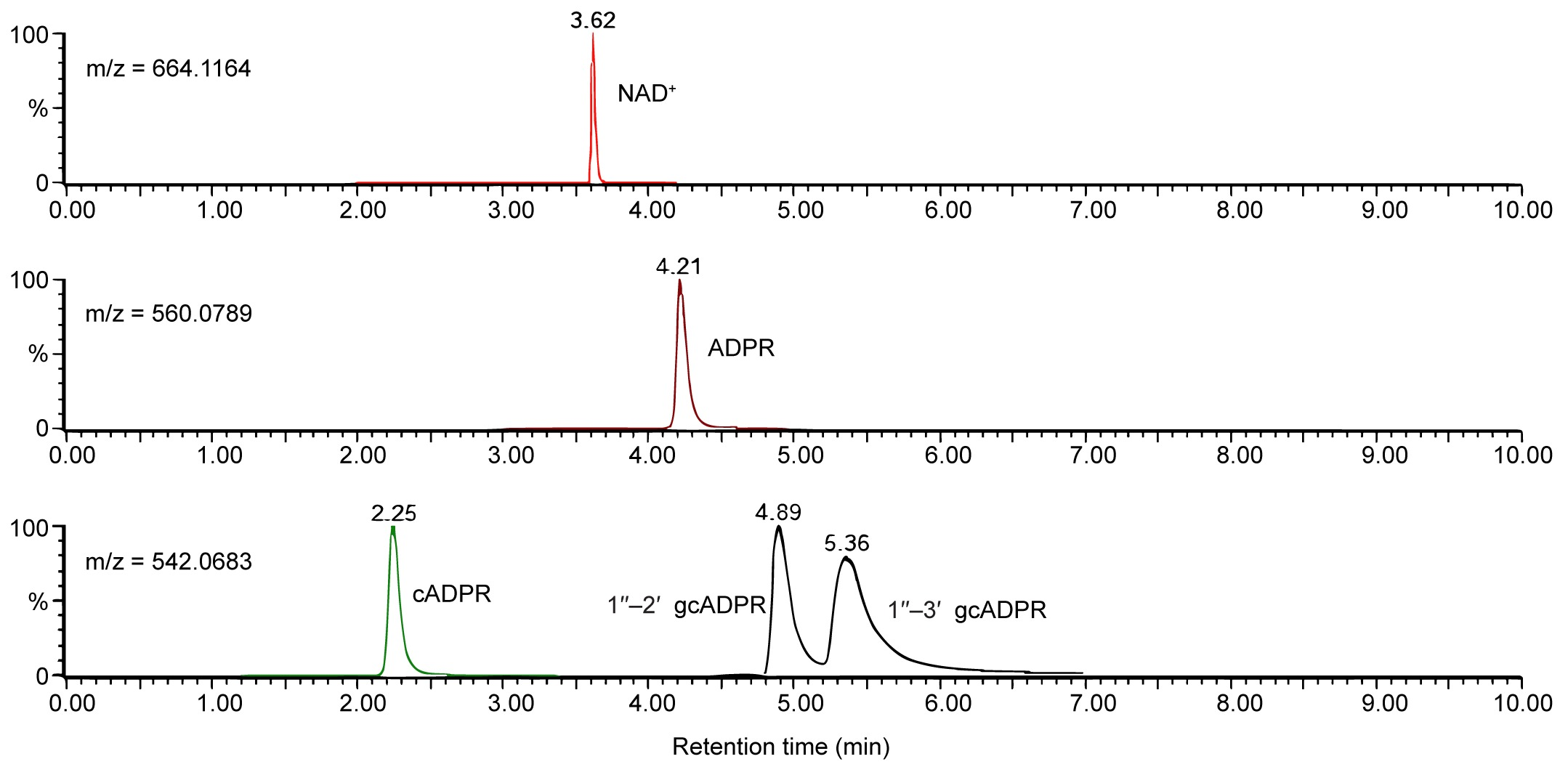

Supplement: S1 Fig — Extracted mass chromatograms of specified ions, detected in a sample containing equal concentration of each standard, demonstrating the difference in retention times. Peak height is normalized to the highest peak in frame. (TIF) [file pone.0302251.s001.tif]

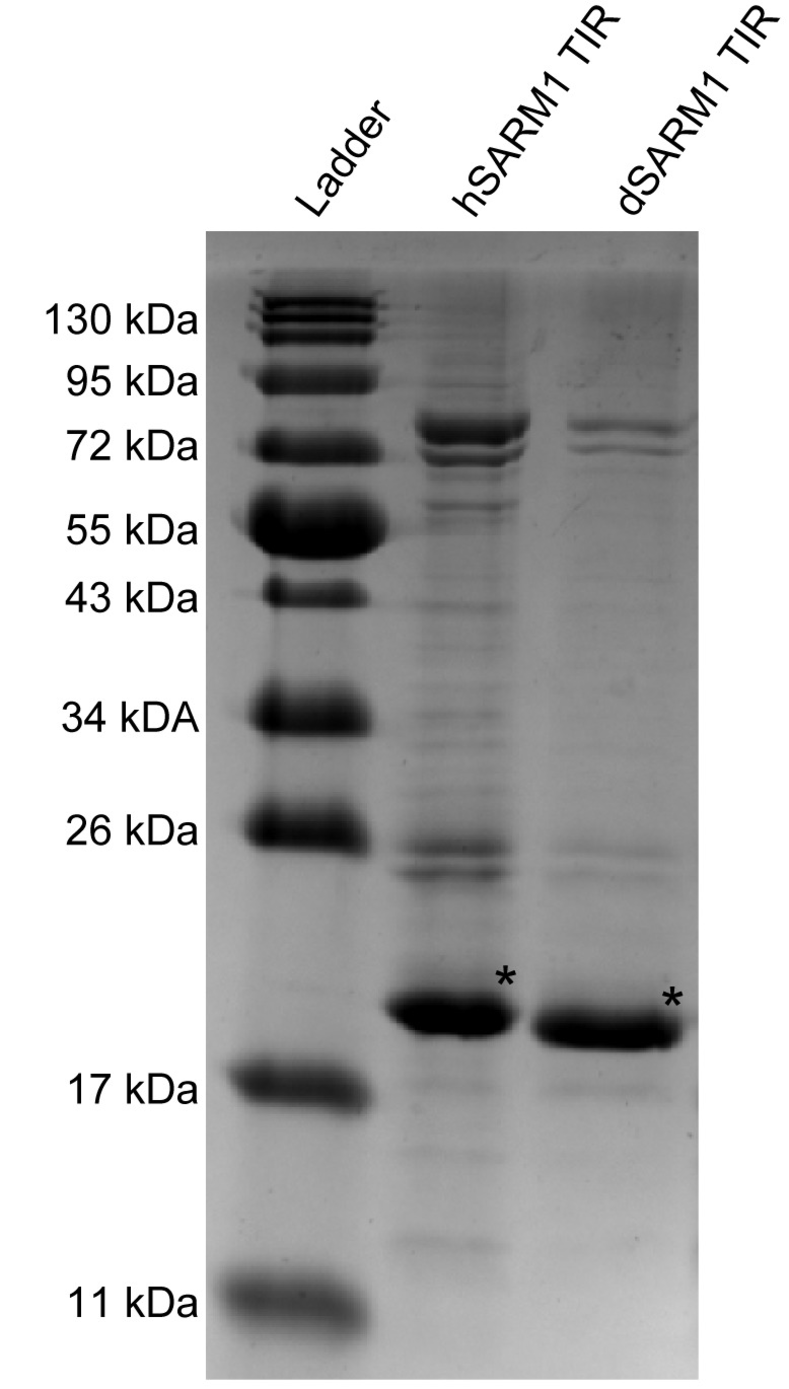

Supplement: S2 Fig — ~2 μg purified protein was separated on a 15% bis-acrylamide SDS gel and sizes were estimated using protein standard. N-terminally 6×His-tagged fusions of human and Drosophila SARM1 TIR have an expected molecular weight of 19 kDa (corresponding bands marked by asterisk). (TIF) [file pone.0302251.s002.tif]

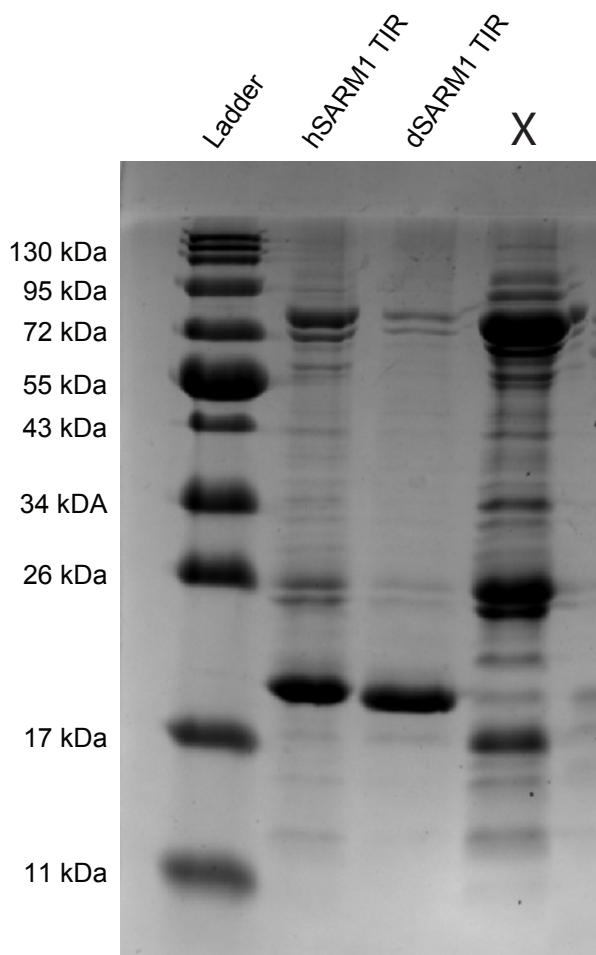

Full SDS-PAGE gel image of Fig S2.

Supplement: S7 File — (PDF) [file pone.0302251.s010.pdf]
