# Supplementary material for: The SARM1 TIR domain produces glycocyclic ADPR molecules as minor products
Source: PLoS One. 2024 Apr 18;19(4):e0302251. doi: 10.1371/journal.pone.0302251 (PMC11025887; doi:10.1371/journal.pone.0302251)
Supplement: Attachment — Submitted filename: PlosOne Review TIR domain SARM1.docx [file pone.0302251.s011.docx]

The manuscript titled “The SARM1 TIR domain produces glycolytic ADPR molecules as minor products” discusses the generation of 1''-2 and 1''-3 glycolytic ribose molecules as subordinate products of TIR domains in both human and Drosophila SARM1. The authors, through well-defined experiments, have preliminarily concluded that these molecules are minor reaction products, yet they also suggest that these byproducts may play a role in SARM1-induced programmed axonal death in animals. However, they have not provided evidence for the latter part. Despite this, the novel findings are noteworthy and could be considered for acceptance once the following concerns are addressed.

1. In an invitro setting, it is unclear whether the 1”-2 and 1”-3 gcADPR molecules (in concentrations ranging from high nanomolar to low micromolar) alone or in combination with ADPR or cADPR induce cell death of cultured axons.
2. The inclusion of a mutated TIR domain protein in the mass spectrometry analysis presented in Figure 2 would enhance the study.
3. An explanation for the increased activity of the Drosophila TIR domain compared to the human TIR domain would be informative.
4. Please include the significance values of the figures.
